# RETRACTION: Effect of Vacuum Sealing Drainage on Healing Time and Inflammation‐Related Indicators in Patients With Soft Tissue Wounds

**DOI:** 10.1111/iwj.70650

**Published:** 2025-04-23

## Abstract

**RETRACTION:** XueX. , LiN. , and RenL. , “Effect of Vacuum Sealing Drainage on Healing Time and Inflammation‐Related Indicators in Patients With Soft Tissue Wounds,” International Wound Journal 18, no. 5 (2021): 639–646, 10.1111/iwj.13565.33786980 PMC8450791

The above article, published online on 30 March 2021, in Wiley Online Library (http://onlinelibrary.wiley.com/), has been retracted by agreement between the journal Editor in Chief, Professor Keith Harding; and John Wiley & Sons, Ltd. Following an investigation by the publisher, all parties have concluded that this article was accepted solely on the basis of a compromised peer review process. In addition, further investigation by the publisher found that the authors provided incomplete ethical approval information for the study. The editors have therefore decided to retract the article. The authors did not respond to our notice regarding the retraction.

**RETRACTION:** X. Xue , N. Li , and L. Ren , “Effect of Vacuum Sealing Drainage on Healing Time and Inflammation‐Related Indicators in Patients With Soft Tissue Wounds,” International Wound Journal 18, no. 5 (2021): 639–646, 10.1111/iwj.13565.33786980 PMC8450791

The above article, published online on 30 March 2021, in Wiley Online Library (http://onlinelibrary.wiley.com/), has been retracted by agreement between the journal Editor in Chief, Professor Keith Harding; and John Wiley & Sons, Ltd. Following an investigation by the publisher, all parties have concluded that this article was accepted solely on the basis of a compromised peer review process. In addition, further investigation by the publisher found that the authors provided incomplete ethical approval information for the study. The editors have therefore decided to retract the article. The authors did not respond to our notice regarding the retraction.

